# The impact of teachers’ perceived competence in information and communication technology usage, and workplace anxiety on well-being, as mediated by emotional exhaustion

**DOI:** 10.3389/fpsyg.2024.1404575

**Published:** 2024-08-06

**Authors:** Bo-Ching Chen, Yu-Tai Wu, Ya-Ting Chuang

**Affiliations:** ^1^Undergraduate Program of Sports Coaching, CTBC Business School, Tainan, Taiwan; ^2^Office of Physical Education, Soochow University, Taipei City, Taiwan; ^3^Department of Education Curriculum & Instruction, National University of Tainan, Tainan, Taiwan

**Keywords:** burnout, COVID-19, mental health, social cognitive theory (SCT), sustainable development goals (SDGs)

## Abstract

**Introduction:**

The 2030 Sustainable Development Goals (SDGs) were adopted by the United Nations in 2015, emphasizing the importance of achieving peace, prosperity, and well-being for all people. With the outbreak of the COVID-19 pandemic, sustainable health has become an important issue. Teachers were forced to adopt distance teaching, necessitating rapid upgrading of their ICT skills and integration into e-learning, which caused tangible and intangible pressures on teachers and impacted their well-being. This study examined the effects of ICT competence on teachers’ workplace anxiety, emotional exhaustion, and well-being during the pandemic from the perspective of Social Cognitive Theory (SCT).

**Methods:**

A quantitative research methodology and a questionnaire survey with a total of 21 questions were used as the primary research design. The snowball method was employed as a sampling method for online questionnaires from September to October 2021. A total of 216 questionnaires were collected, of which four incomplete questionnaires were excluded, leaving 212 valid questionnaires, with a valid questionnaire recovery rate of 98.1%. The valid questionnaires were analyzed using Smart Pls 4.0 Partial Least Square Method Structural Equation Modeling (PLS-SEM).

**Results:**

The study found that teachers’ ICT competence could significantly reduce emotional exhaustion and enhance teachers’ well-being. However, there was no significant effect on workplace anxiety. Additionally, well-being was not directly affected by workplace anxiety, and teachers’ well-being needs to be mediated by emotional exhaustion to be indirectly affected. Emotional exhaustion plays an important mediating role between teachers’ ICT competence and workplace stress, both of which are important mediators of well-being.

**Discussion:**

From a practical point of view, to achieve the Sustainable Development Goals (SDGs) 2030, it is ideal to have good health and well-being for the whole person. This study facilitates the development of strategies to improve the well-being of teachers, which provides an empirical basis for the enhancement of mental health and well-being of educators.

## Introduction

Since the 2019 COVID-19 outbreak, the global community has been hit like never before (Wang and Sun, 2022; Hamid et al., 2023). In the field of education, many educational institutions suspended face-to-face teaching for pandemic prevention and chose to use internet technology for distance learning, beginning a period of emergency distance education (Hodges et al., 2020). As schools and educational institutions were forced to adopt new modes of teaching online and working remotely, teachers who were familiar with face-to-face teaching had to quickly adapt to the environment of integrating Information and Communication Technology (ICT) into online teaching (Mahlangu and Makwasha, 2023; Rojas Briñez et al., 2023). However, due to insufficient preparation for online teaching, many teachers faced a range of challenges during this period, such as managing virtual courses, maintaining a stable internet connection, and their own ICT skills deficits. In other words, the impact of the pandemic has rewritten the teaching scene and made teachers’ roles and responsibilities more complex (Alakrash and Razak, 2022; Wang and Sun, 2022). As a result, teachers were forced to suddenly acquire a high level of ICT competence and knowledge in order to cope with new forms of work pressure and challenges. In this environment, teachers are constantly battling against stress, resulting in negative emotions such as burnout and emotional exhaustion (Bahamondes-Rosado et al., 2023; Cornes et al., 2023; O’Flaherty and McCormack, 2023).

According to the Social Cognitive Theory (SCT) proposed by Bandura (1986, 1997), human behavior is based on the interplay of personal factors, environmental influences, and purposeful behavior (Bandura, 2001, 2009). Therefore, self-efficacy plays an important role in this theory, representing a person’s confidence in his or her ability to accomplish a particular task (Bandura, 1986). In addition, observational learning, or “vicarious learning,” describes how people learn or modify behavior by observing and imitating others and evaluating the results of the observed behavior (Bandura, 1986). In the online teaching environment during the pandemic, many teachers were faced with the challenge of teaching entirely at a distance for the first time, which may have triggered concerns about the use of technology, declining student engagement, and interaction with students at a distance. According to Bandura’s (2001, 2009) Social Cognitive Theory (SCT), these new challenges can be understood in terms of the interactions between the individual, the environment and behavior. In recent years, psychological, educational, and vocational research on ICT has adopted the SCT perspective to explore the psychological mechanisms associated with users’ use of technology (Abraham et al., 2022; Farmania et al., 2022; Gözüm et al., 2023; Suryanto et al., 2022; Huang and Zeng, 2023). Therefore, when teachers feel they are ready to cope with Information Communication Technology (ICT) challenges, their anxiety and emotional fatigue may decrease, which in turn may increase their well-being. They may be able to gain strategies and confidence in the process by observing how their colleagues or other educators are coping with ICT challenges but may also experience increased stress by observing how others are working (Stan, 2022; Lee et al., 2023; Rastegar and Rahimi, 2023).

Finally, in 2015, the United Nations adopted the 2030 Sustainable Development Goals (SDGs), which seek to achieve peace, prosperity and well-being for all by 2030. In the third goal of the SDGs (SDG3), specific targets emphasize the importance of good health and well-being, environmental sustainability, health and happiness (Sweileh, 2020). However, preventive measures such as segregation and social distancing during the COVID-19 pandemic had varying degrees of impact on the mental health of the population and posed a significant challenge to the achievement of these SDGs. Health and wellbeing are seen as key elements of sustainable development, but in recent years, due to pandemic-induced changes in the educational paradigm, the traditional classroom model has been overthrown and replaced by online teaching and remote working, which integrates ICT with online teaching and learning, placing greater demands on teachers’ ICT competencies, and this change has forced teachers to face greater stress and has had a significant impact (Cornes et al., 2023; O’Flaherty and McCormack, 2023; Pankowski et al., 2023; Rastegar and Rahimi, 2023; Solís et al., 2023).

Despite the unprecedented challenges faced by teachers during the COVID-19 pandemic, prior to the pandemic, numerous studies have shown that teachers’ easy access to information and technological competencies can increase their self-efficacy and job satisfaction (Hampton et al., 2020; Baroudi and Shaya, 2022). Teachers who were able to flexibly integrate technology into their teaching activities not only improve their teaching outcomes and reduce their workload, but also have higher job satisfaction, increasing their motivation and dedication, thus contributing to the achievement of the United Nations’ Sustainable Development Goals (Barba-Sánchez et al., 2022). However, the changes during the COVID-19 pandemic did result in little exploration of the associations between teachers’ perceived ICT competence, workplace anxiety, emotional exhaustion, and well-being. Therefore, this study, based on social cognitive theory and research hypotheses, aims to answer the following four main research questions:

1. How do teachers’ Information and Communication Technology (ICT) competencies affect their workplace anxiety, emotional exhaustion and well-being?
2. How does teachers’ workplace anxiety affect their emotional exhaustion and sense of well-being?
3. How does teachers’ emotional exhaustion affect their sense of well-being?
4. How do teachers’ ICT competence and workplace anxiety indirectly affect their well-being through emotional exhaustion?

Finally, this study investigates how rapidly the Taiwanese education system had shifted to online teaching and learning during the COVID-19 pandemic in 2021. This period was not only a critical time for teachers to adapt to the challenges of information technology (ICT) competence, but also a time of rapidly increasing responsibilities and psychological stress. By exploring the interrelationships between teachers’ ICT competence, workplace anxiety, emotional exhaustion and well-being, this study provides insights into how teachers are coping with these changes and their impact on well-being. With the advent of the post-pandemic era, online teaching has become an essential skill for teachers, a change that is not only crucial for their personal development, but also a key factor in moving the entire education system towards sustainability. Therefore, this study is crucial for current and future education policy making, not only providing valuable historical references for responding to global crises, but also contributing to the realization of the United Nations’ Sustainable Development Goals (SDGs) for education and health.

## Literature review

### Social cognitive theory

Bandura (1977) Social Learning Theory (SLT) emphasizes observing and modeling others’ behaviors, attitudes, and emotional responses. The theory assumes that most human behaviors are learned through modeling observations. SLT explains human behaviors through the continuous interactions between cognition, behavior, and environmental influences. Social Cognitive Theory (SCT) is derived from Social Learning Theory (SLT) and is relevant to healthy communication mainly because it involves cognitive and affective events, environmental factors, and behaviors (Mobley and Sandoval, 2008).

The key structures of social cognitive theory include observation learning, reinforcement, self-control, and self-efficacy. According to SCT, there is a continuous interaction between environment, cognition, and behavior, and it is constantly changing. The use of distance education as a social environment surged due to the emergency of the pandemic and had an impact on teachers’ cognition and teaching behavior. The impact of the social environment on an individual’s cognition and behavior may be either positive or negative (Ortan et al., 2021). During the pandemic, when teachers observed peers with higher ICT competence, they would use those peers as a reference point. Based on the theory, this study hypothesized that teachers with insufficient ICT competence would have a negative evaluation of their own competence after observing their peers’ high performance (high ICT competence) and comparing them to themselves, which may have led to negative emotions such as stress, anxiety, and low self-efficacy. These negative emotions would negatively affect their subjective well-being, possibly leading to negative emotions such as stress, anxiety, and low self-efficacy, which may also negatively affect their subjective well-being. Thus, the associations between ICT competence, workplace anxiety, emotional exhaustion, and teachers’ well-being in online teaching environments constitute a complex network of relationships.

### Information and communication technology competence (ICT)

ICT competence is defined as an individual’s knowledge, skills and abilities in the field of Information and Communication Technology, which covers the ability to use computers, software, networks and other digital tools to work, learn and live effectively (Oberländer et al., 2020). Educators face many challenges in developing students’ digital competence. In their own professional practice, teachers must not only use existing and emerging digital resources, but must also think about how to enable students to use technology effectively. This involves the digital competence needs of students and how teachers specifically instruct, design learning, organize lessons, and conduct assessments. The aim is to make better use of digital resources to support student learning (Falloon, 2020). The development of ICT capabilities will collectively drive the digital transformation of education and ensure that teachers remain professionally competitive and happy in the digital age (OECD, 2019). In the wake of the COVID-19 pandemic, many teachers have shared their difficulties in teaching online. Among them, the inability to observe students’ facial expressions, their attention span, and their interest in particular subject matter has created many obstacles to teaching. Furthermore, most teachers had little or no experience teaching online prior to the outbreak, so they had limited knowledge of online teaching tools. This made them more likely to feel stress and anxiety while teaching, and many cited technologies as the main cause of their teaching difficulties (Chandwani et al., 2021). Therefore, teachers’ self-efficacy has become a crucial factor in the current teaching and learning environment because it directly affects teachers’ willingness to use technology for teaching and its effectiveness. The so-called self-efficacy actually refers to teachers’ confidence in their ability to utilize ICT. In other words, when teachers have a higher sense of self-efficacy, they are likely to be more motivated to use technology tools in teaching and are more likely to achieve the desired results (Huang et al., 2022; Afari et al., 2023).

The pace of ICT implementation in education increased significantly due to the urgent need for digitization in the society during the pandemic. However, these changes not only increased teachers’ workplace anxiety (Jurek et al., 2021), but also highlighted the diversity of digital tools. While these tools are undoubtedly beneficial to teaching and learning, they also have a negative impact in terms of fatigue and stress, especially for teachers in primary and secondary schools in the country. These teachers are under tremendous pressure to make this change, which in turn deeply affects their quality of life (Francom et al., 2021; Idoiaga Mondragon et al., 2021). Based on the above background, from the perspective of social cognitive theory, where people observe others’ learning and imitation behaviors, during the pandemic, teachers may observe the online teaching effectiveness of other colleagues and their own perceptions of their own ICT abilities, resulting in self-efficacy and workplace anxiety, which in turn affects their level of anxiety and emotional exhaustion at work and their physical and mental health. First, when teachers have a strong sense of self-efficacy, their confidence in their ability to use ICT for teaching and learning increases. Such self-confidence not only helps them to utilize ICT tools more effectively, but also reduces workplace anxiety. Secondly, the more ICT competent teachers are, the less psychological and emotional burden they may experience in online teaching, which has a negative effect on their emotional exhaustion. Lastly, when teachers have high self-efficacy as a result of their ICT competence, their job fulfillment and satisfaction may increase, which in turn reduces their emotional exhaustion and increases their overall well-being. Based on this reasoning, the study proposed the following hypotheses:

*Hypothesis 1*: Teachers’ ICT competence has a negative effect on their workplace anxiety.

*Hypothesis 2*: Teachers' ICT competence has a negative effect on their emotional exhaustion.

*Hypothesis 3*: Teachers' ICT competence has a positive effect on their well-being.

### Workplace anxiety

During the pandemic, a growing number of teachers suffered from high levels of work stress, anxiety, depression and burnout (Ozoemena et al., 2021). The underlying causes of anxiety at work vary from person to person, and for some people, excessively long working hours, high stress levels, lack of support from coworkers, and related factors may lead to anxiety at work (van Steijn et al., 2019). A stressful work environment increases teachers’ occupational stress, causing widespread illnesses. Teachers have more physical conditions including health problems (i.e., hypertension, headaches, and cardiovascular disease) and mental health problems (i.e., anxiety, depression, and physical illnesses) than other occupations, and have a lower quality of life and shorter life expectancy (Lainidi et al., 2023). There is a link between workplace anxiety, emotional exhaustion and mental health problems, which can have a detrimental effect on an individual’s sense of well-being (Cheng and McCarthy, 2018). The COVID-19 pandemic has had a profound impact on most workplaces, especially the teaching environment. Teaching situations are further complicated by the lack of necessary resources, such as adequate personal protective equipment, teaching and learning materials, and basic technological equipment (Agormedah et al., 2020; Hagan et al., 2022; Hershkovitz et al., 2023). In such contexts, teachers have to face tremendous psychological stress, especially when they are trying to achieve their teaching and other academic goals under the influence of the pandemic. These challenges stem in part from the context-specific constraints imposed by the pandemic (Gutentag and Asterhan, 2022; Pankowski et al., 2023). Based on the above, when teachers are exposed to a prolonged period of anxiety in the work environment, it will increase their professional stress and may cause them to fall into a state of emotional exhaustion, which also means that an anxious environment will be more likely to cause damage to their mental health or to increase the risk of illness. Accordingly, the following hypotheses were formed:

*Hypothesis 4*: Teachers' workplace anxiety positively affects their emotional exhaustion.

*Hypothesis 5*: Teachers' workplace anxiety negatively affects their Well-Being.

### Emotional exhaustion

The COVID-19 pandemic has had a dramatic impact on schooling, with a rapid shift from traditional face-to-face learning to online modes of learning. While some teachers and students adapted quickly and effectively to this change, many still faced difficulties such as feelings of isolation, burnout, and loss of a sense of belonging in traditional face-to-face classrooms (Parte and Herrador-Alcaide, 2021). In particular, many teachers described facing significant challenges and exhaustion during the pandemic, such as coping with sudden school openings and closures, the ongoing risk of contracting the virus, the constant switching between online and physical classrooms, and the continuous stress of teaching online (Gadermann et al., 2023). Emotional exhaustion is one of the symptoms of burnout, which is particularly pronounced among teachers, negatively affecting their career development, and the sense of depletion of emotional and psychological resources negatively affects their well-being (Ortan et al., 2021; Fox and Walter, 2022; Padmanabhanunni and Pretorius, 2023). According to the above studies, the following hypothesis was formulated for this study:

*Hypothesis 6*: Teachers' emotional exhaustion will negatively affect their well-being.

### Well-being

The well-being of teachers is affected by multiple factors which consist of teachers’ personal characteristics and the organizational environment. Personal factors comprise the teacher’s style of coping, personality traits, and their self-confidence, which influence how they feel about their life and work. At the same time, organizational factors play an important role; these factors include educational environment quality, the clarity of role descriptions, and the influence of leadership styles. Additionally, social factors have an impact on educators’ well-being; they include socio-cultural background, economic status, and so on (Berger et al., 2022). With the increasing awareness of emotional stress, a growing number of studies have begun to examine various aspects of teachers’ mental health. These studies have covered aspects such as teachers’ cognitive work stress, children’s behavioral challenges, and so on, and have examined the association between these factors and teachers’ mental health, occupational stress, and well-being (Kwon et al., 2022). Teachers with high self-efficacy may contribute to their well-being due to less stress in as teachers may feel less anxiety and emotional exhaustion when facing ICT challenges (Billett et al., 2022; Fox and Walter, 2022). In contrast, self-efficacy was found to be negatively associated with emotional exhaustion because autonomy meant that teachers would not be instructed to use teaching methods with which they were unfamiliar, which could require additional time for preparation, potentially decreasing teachers’ well-being and affecting their overall emotional state (Hascher et al., 2021). Finally, summarizing the relationships between the above variables, this study aimed to further understand the sources of these stressors based on a social cognitive theory perspective. Teachers in such an environment may adjust their approach by observing their colleagues’ online teaching strategies, but this may also affect their level of anxiety and emotional exhaustion (Soto et al., 2021). In addition, due to the rapid evolution of ICT, teachers face increasing work challenges such as fragmentation, high levels of interference and complex tasks (Pfaffinger et al., 2020; Rabaglietti et al., 2021). This constant stress may not only trigger fatigue and burnout, but may also lead to other health problems, which in turn could affect their overall teaching effectiveness and quality of life (Zhao et al., 2022). Summarizing the above hypotheses, we can infer that high ICT competence has a positive impact on teachers’ well-being, and that increased self-efficacy in ICT competence may help to reduce teachers’ emotional exhaustion, which in turn increases their well-being. In addition, workplace anxiety may have a positive effect on emotional exhaustion and reduce their well-being. Based on the above, the following two mediated effect hypotheses were proposed in this study:

*Hypothesis 7*: Teacher ICT competence negatively affects well-being through emotional exhaustion.

*Hypothesis 8*: Workplace anxiety negatively affects well-being through emotional exhaustion.

## Method

**Figure 1:**
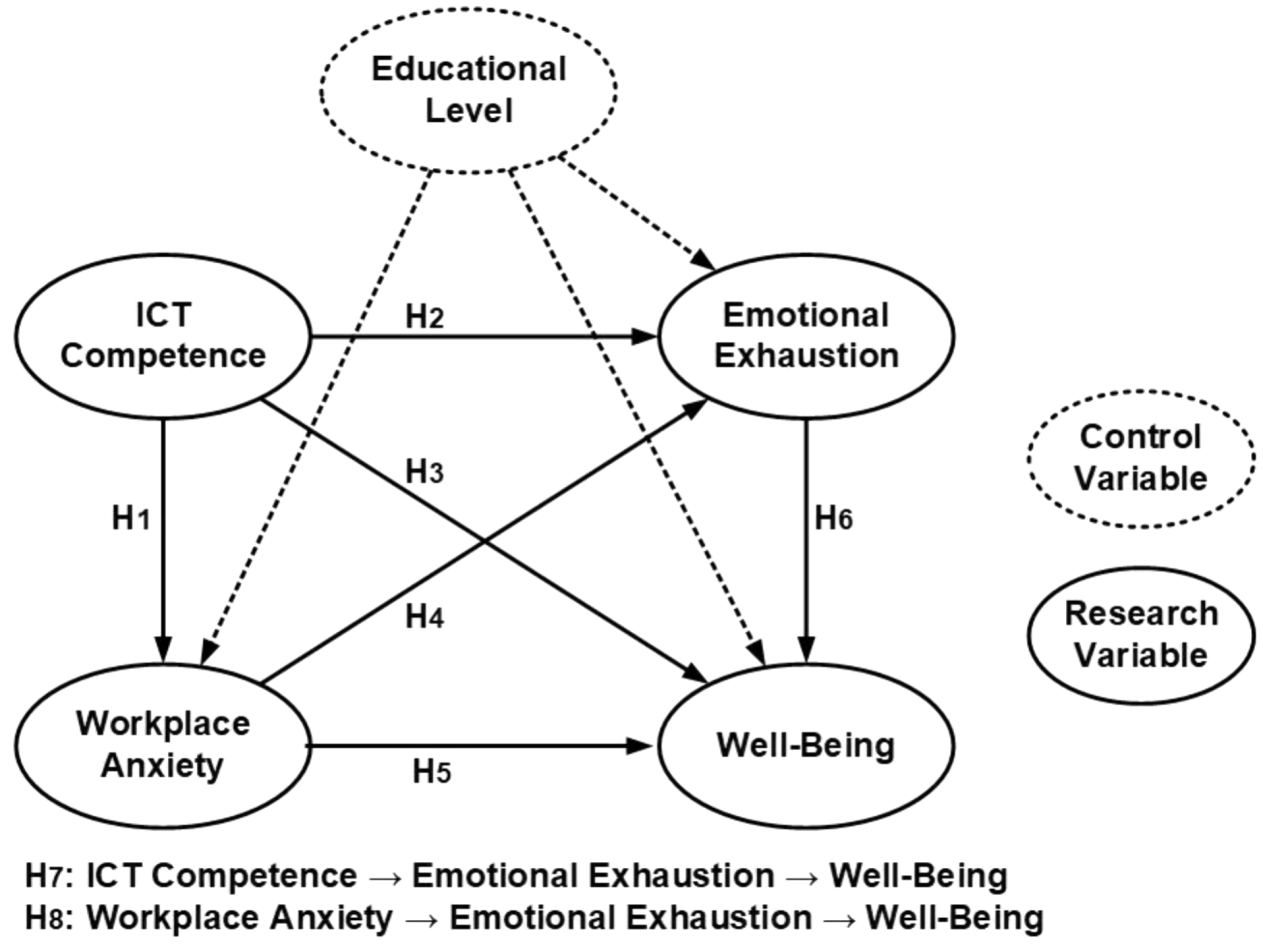
Research framework.

According to the background motivation of this study, after confirming the topic, the relationships among ICT Competence (ICT), Workplace Anxiety (WA), Emotional Exhaustion (EE), and Well-Being (WB) were explored in the literature, and a hypothetical model was constructed from the perspective of Social Cognitive Theory. Finally, in order to cope with the specificity of different levels in the education field, this study controlled for the dummy variable according to the different levels of schools (elementary, middle, and high school) in which the teachers were located. See the model diagram below shown in Figure 1 and See Table 1 for supporting literature.

**Table 1:** Hypotheses and related empirical studies.

Hypotheses	Variable relationship	Theoretical and empirical studies
H_1_	ICT → WA	Chandwani et al. (2021), Idoiaga Mondragon et al. (2021), and Soto et al. (2021)
H_2_	ICT → EE	Bahamondes-Rosado et al. (2023), Chandwani et al. (2021), and Jurek et al. (2021)
H_3_	ICT → WB	Fox and Walter (2022) and Pfaffinger et al. (2020)
H_4_	WA → EE	Agormedah et al. (2020), Fox and Walter (2022), Hagan et al. (2022), and Hagan et al. (2022)
H_5_	WA → WB	Lainidi et al. (2023) and van Steijn et al. (2019)
H_6_	EE → WB	Francom et al. (2021), Fox and Walter (2022), and Padmanabhanunni and Pretorius (2023)
H_7_	ICT → EE → WB	Gutentag and Asterhan (2022) and Huang et al. (2022)
H_8_	WA → EE → WB	Pfaffinger et al. (2020) and Siddiqui et al. (2023)

IC, ICT competence; EE, emotional exhaustion; WB, well-being; WA, workplace anxiety.

### Research design

This study was conducted during the epidemic period in Taiwan from September to October 2021, when primary and secondary schools fully switched to online distance learning. This cross-sectional study investigated all teachers’ online-only teaching activities during that period. The questionnaire consisted of two parts: the first part consisted of 21 questions on four core variables considering ICT Competence, workplace anxiety, emotional exhaustion, and well-being, based on previous questionnaires with good reliability and taking into account the contextual background and purpose of the study; The second section collected information on the background variables, including age, education, school level of employment, school attribute, school location, school size, employment status, and the number of questions on the four core variables. The following seven items are included, and years of service.

To ensure the reliability and validity of the results of this study, data were analyzed using Partial Least Squares Structural Equation Modeling (PLS-SEM), which is particularly suitable for exploratory theoretical modeling and verification of inferred causal relationships. It has been suggested that the sample size for PLS analysis should be at least ten times larger than that of all the questions measured (Gefen et al., 2011). Considering the 21 measurement questions involved in this study, in other words, at least 210 samples are needed to ensure the results of the statistical analysis. Based on the above, the limitations of physical contact and the reduced accessibility of participants during the COVID-19 pandemic, PLS-SEM is an effective analytical method due to its high adaptability to smaller sample sizes. This facilitates effective data analysis under various constraints, thus ensuring the quality and progress of the study.

**Figure 2:**
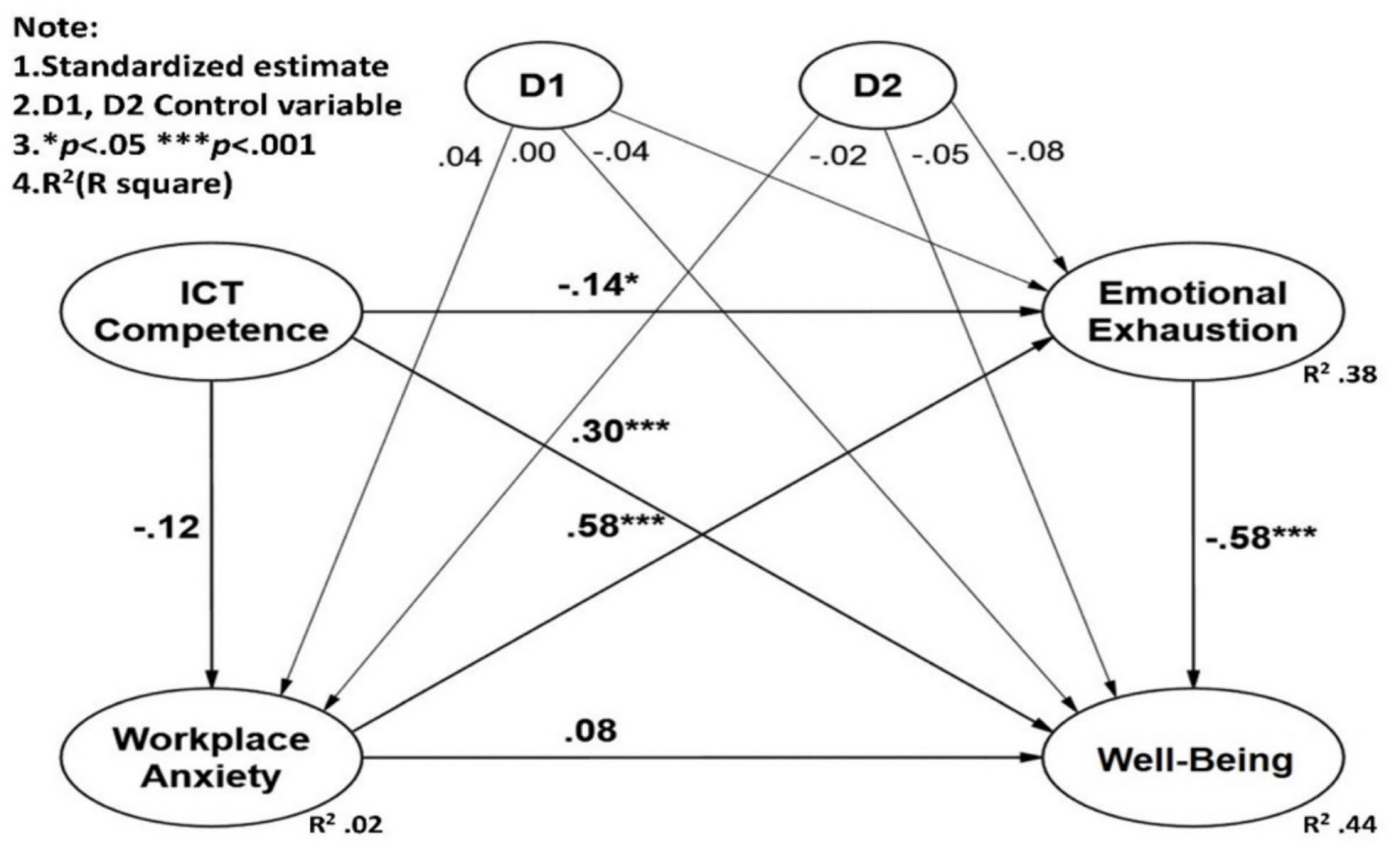
Theoretical model parameter estimation.

### Participants

To ensure the good quality of questionnaire data received for this study, an online questionnaire collection method was adopted, and the Google Forms platform was chosen to minimize invalid responses due to its convenience and omission reminder function. During the pandemic period, this remote online method avoided unnecessary face-to-face contact and facilitated the free participation of the respondents.

To increase the sample diversity, local academic directors were contacted via email and LINE, a social media platform, and supported by some of them, who assisted in distributing the questionnaire links to their respective groups of teachers. In addition, acquaintances were encouraged to promote the survey to their teacher friends to further widen the scope of participation. In terms of research ethics, the study strictly followed the guidelines of the Declaration of Helsinki. All participants were informed of the purpose of the study, confidentiality, anonymity, and their right to participate before completing the questionnaire to ensure the security of their information and to respect the principle of voluntary participation. All feedback from participants was handled anonymously and used only for academic research purposes. As a token of appreciation to the participating teachers, small gifts were provided as an incentive to promote high quality data collection.

### Research instruments and methodology

The data obtained from the study questionnaires were analyzed using the least squares method PLS-SEM 4.0 package, which has been widely used in many fields (Hair et al., 2009; Khan et al., 2019; Sarstedt et al., 2020). In addition to the fact that the data can be distributed non-constant to calculate the advantages of complex models using smaller sample sizes. The method is also effective in terms of estimating the mediator model in one model at a time, which can significantly increase the statistical validity compared with the traditional segmented path analysis (Joreskog, 1982; Hair et al., 2009; Shiau et al., 2019; Sarstedt et al., 2020; Cheah et al., 2021). Based on the above, analysis was performed using Smart PLS 4.0 in accordance with the purpose of the study and in order to ensure the correctness of the statistical analysis. The questionnaire is explained in detail below.

#### Questionnaire

This study was designed according to the variables in the above research framework, and the questionnaire design was divided into two parts; the first part was for the various dimensions of the study, and the second part was for the demographic background variables. There are four dimensions in this study, with a total of 21 observation questions. Each dimension is described as follows.

Please refer to the Appendix for a complete list of measurement topics in this study.

#### IC: ICT competence

Teachers’ perceived ICT competence is a variable that reflects individual teachers’ perceptions of their own ICT-related knowledge and skills. In this study, the Teacher Perceived ICT Competence Questionnaire was based on the global Program for International Student Assessment (PISA), which is administered by the Organization for Economic Cooperation and Development (OECD) every 3 years. The participants of the PISA survey are from the Organization for Economic Cooperation and Development (OECD) countries. The ICT competency indicator questionnaire was designed according to the program’s 2015 and 2018 phases (Hori and Fujii, 2021; Hu and Yu, 2021). In the past, some scholars used PISA data to validate the Perceived ICT competence scale in 16 countries, and the McDonald’s reliability index ranged from 0.907 to 0.939, which means that the questionnaire has a very good reliability (Ma and Qin, 2021). This study used a 7-point Likert scale ranging from 7, *strongly agree,* to 1, *strongly disagree*, with higher scores representing higher teachers’ perceived ICT competence; α = 0.88. with item example: ‘I feel comfortable using digital devices that I am less familiar with.,” “If my friends and relatives want to buy new devices or applications, I can give them advice.” and “If my friends and relatives want to buy new devices or applications, I can give them advice”.

#### WA: workplace anxiety

Workplace Anxiety (WA) mainly refers to teachers’ feelings of nervousness and anxiety in order to accomplish teaching-related tasks, which is a variable that can be affected by individual differences and school contexts. This study referred to Workplace Anxiety Items, with a good reliability of (α = 0.94) (McCarthy and Goffin, 2004; McCarthy et al., 2016). According to the research purpose, from which five questions were deemed to be more suitable for teachers’ teaching place situation questions. A 7-point Likert scale was used, from 7 points for *strongly agree* to 1 point for *strongly disagree*, where a higher score indicates higher workplace anxiety. The α = 0.91. item examples were “I am overwhelmed by thoughts of doing poorly at work,” “I worry that my work performance will be lower than that of others at work.” and “I feel nervous and apprehensive about not being able to meet performance targets”.

#### EE: emotional exhaustion

The Emotional Exhaustion Scale (EES) used in this study was based on the Emotional Exhaustion construct of the Maslach Burnout Inventory, which is widely used and has been found to be reliable (Maslach and Jackson, 1981; Aryee et al., 2008). In recent years, this scale has shown good reliability and validity in applied studies in different countries, such as in Italy among elementary school teachers with an alpha value of 0.85 (Pellerone et al., 2020) and in Portugal among teachers in higher education institutions with an alpha value of 0.92 (Teles et al., 2020). The scale consists of six questions on a 7-point Likert scale ranging from 1 (completely disagree) to 7 (completely agree), with higher scores indicating higher levels of emotional exhaustion. In this study, the alpha value of the scale was 0.91. Sample questions included “My job makes me exhausted.,” “After I finish my job, I feel exhausted.” and “When I think of the whole day’s work in When I think of the whole day’s work in the morning, I feel tired.

#### WB: well-being

To assess teachers’ perceived health and well-being, this study drew on the five-item World Health Organization Well-Being Index (WHO-5) developed by the World Health Organization, the scale was also validated in Taiwan, with good internal consistency and an alpha value of 0.94 (Lin et al., 2013; Topp et al., 2015), which has now been translated into at least 30 languages and has been used in many studies around the world since it was first published in 1998. It is one of the most widely used questionnaires for assessing subjective psychological well-being. The scale consists of five questions and was administered on a 7-point Likert scale ranging from 7, *strongly agree*, to 1, *strongly disagree*, with higher scores representing higher subjective psychological well-being; α = 0.92 in this study. Examples of items were “I have felt cheerful and in good spirits” and “I have felt calm and relaxed.” “I have felt active and vigorous”.

## Result

In this study, during the pandemic period from September 19 to October 21, 2021, the snowball method was used to facilitate sampling of the questionnaire survey through teachers, school directors, relatives and friends who were familiar with the education sector, using the Google Forms web-based questionnaire system. A total of 216 samples were recovered, from which four incomplete questionnaires were excluded, leaving a total of 212 valid questionnaires, which represents a valid questionnaire recovery rate of 98.1%. Among the recovered questionnaires, 127 female teachers accounted for 59.9%, while 85 male teachers accounted for 40.1%. The age of 41–50 years old was the most, totaling 108, accounting for 50.9%; 31–40 years old was the next with 58 teachers, accounting for 27.4%, and the third was 51–60 years old, totaling 35, accounting for 16.5%. As for school level, there were 57 teachers at each of the elementary school and middle school stages, accounting for 26.9% each, 64 at the high school level, accounting for 30.2%, and 91 at the senior high school level (including senior high schools, vocational high schools, middle schools, complete middle schools, and comprehensive high schools), accounting for 42.9%. The largest concentration of schools was in southern Taiwan, with 150 teachers, accounting for 70.8% of the total, followed by 36 teachers in northern Taiwan, accounting for 16.5%, and 26 teachers in central Taiwan, accounting for 12.3% of the total. For more details, please see Table 2.

**Table 2:** Background information of subjects (*N* = 212).

Background	*N*	%		Background	*N*	%
**1. Gender:**				**6. School location**		
(1) Female	127	59.9		(1) North Taiwan	35	16.5
(2) Male	85	40.1		(2) Central Taiwan	26	12.3
**2. Age**				(3) South Taiwan	150	70.8
(1) 21 ~ 30 years	10	4.7		(4) East Taiwan	1	0.5
(2) 31 ~ 40 years	58	27.4		**7. School size:**		
(3) 41 ~ 50 years	108	50.9		(1) 12 classes and below	34	16.0
(4) 51 ~ 60 years	35	16.5		(2) 13 ~ 24 classes	40	18.9
(5) over 60 years	1	0.5		(3) 25 classes and above	138	65.1
**3. Education:**				**8. Employment status**		
(1) University	48	22.6		(1) Part-time	9	4.3
(2) Masters	141	66.5		(2) Substitute	17	8.0
(3) PhD	23	10.8		(3) Full-Time	186	87.7
**4. School level of employment:**				**9. Years of Service**		
(1) Primary	57	26.9		(1) 1 ~ 10 Years	54	25.5
(2) Middle School	64	30.2		(2) 11 ~ 20 Years	101	47.6
(3) High School, Vocational High, Middle School, Complete Middle School, Comprehensive High School	91	42.9		(3) 21 ~ 30 Years	44	20.8
				(4) 30 and above	13	6.1
					
**5. School attribute:**						
(1) Public	200	94.3				
(2) Private	12	5.7				

### Data analysis

In this study, the Smart PLS version 4.0.9.6 software was used to analyze the data using Partial Least Squares Structural Equation Modeling (PLS-SEM), and the data were checked to ensure the quality before the formal analysis.

This study followed Anderson and Gerbing’s (1988) recommendation that a complete structural equation modeling analysis should be based on a two-step criterion. In the first stage, measurement modeling is conducted to verify the reliability and validity of the questionnaire. In the second stage, the overall modeling is conducted to test the research hypotheses.

Referring to Table 3, the recovered samples in this study were based on four latent dimensions: ICT Competence with five questions from ICT1 to ICT5, with the mean ranging from 4.76 to 5.96 and the standard deviation ranging from 1.05 to 1.51; Emotional Exhaustion with six questions from EE1 to EE6, with the mean ranging from 2.71 to 4.16 and the standard deviation ranging from 1.48 to 1.72; Well-Being with questions from WB1 to WB5, with means of 5.09 to 5.45 and standard deviations of 1.14 to 1.34; and Workplace Anxiety with five questions from WA1 to WA5, with means of 3.87 to 4.40 and standard deviations of 1.62 to 1.88. The Kurtosis of all the questions in all the constructs ranged from −1.19 to 2.01, and the standard deviations of the questions were between −1.19 and 2.01, respectively. The Skewness ranged from −1.12 to 1.00. These results conformed to the absolute value of Kurtosis <7 and the absolute value of Skewness <2 as proposed by Kline (2005), and conformed to the criteria of the statistical single-variable normative assignment. In addition, the highest loading value of each question fell in its potential configuration, which is in line with the recommendation of scholars that the highest loading of each question should be in the desired configuration (Chin, 1998; Grégoire and Fisher, 2006).

**Table 3:** Item analysis.

Items	Mean (SD)	Kurtosis	Skewness	Cross loadings			
				1. ICT	2. EE	3. WB	4. WA
1. ICT: ICT competence							
ICT1	4.76(1.49)	−0.55	−0.50	**0.74**	−0.20	0.30	−0.18
ICT2	4.68(1.51)	−0.53	−0.38	**0.84**	−0.15	0.29	−0.10
ICT3	5.96(1.05)	1.32	−1.12	**0.73**	−0.22	0.36	−0.04
ICT4	5.30(1.29)	0.53	−0.78	**0.90**	−0.16	0.37	−0.10
ICT5	4.95(1.39)	−0.09	−0.63	**0.87**	−0.13	0.33	−0.09
2. EE: emotional exhaustion							
EE1	3.65(1.63)	−0.74	0.22	−0.18	**0.81**	−0.51	−0.18
EE2	4.16(1.72)	−1.00	−0.11	−0.18	**0.77**	−0.49	−0.18
EE3	3.31(1.58)	−0.49	0.44	−0.19	**0.88**	−0.54	−0.19
EE4	3.01(1.51)	−0.08	0.61	−0.16	**0.91**	−0.61	−0.16
EE5	2.74(1.48)	0.38	0.85	−0.18	**0.82**	−0.36	−0.18
EE6	2.71(1.48)	0.67	1.00	−0.17	**0.77**	−0.39	−0.17
3. WB: well-being							
WB1	5.45(1.15)	2.01	−1.09	0.33	−0.51	**0.89**	−0.24
WB2	5.40(1.16)	1.74	−1.06	0.32	−0.55	**0.91**	−0.28
WB3	5.35(1.15)	1.15	−0.86	0.33	−0.58	**0.93**	−0.29
WB4	5.09(1.34)	0.39	−0.73	0.36	−0.48	**0.83**	−0.26
WB5	5.43(1.14)	0.99	−0.76	0.44	−0.46	**0.82**	−0.24
4. WA: workplace anxiety							
WA1	3.87(1.66)	−0.99	0.15	−0.17	0.52	−0.30	**0.83**
WA2	4.03(1.83)	−1.19	−0.02	−0.10	0.53	−0.25	**0.91**
WA3	4.40(1.62)	−0.85	−0.32	−0.11	0.53	−0.27	**0.84**
WA4	4.26(1.88)	−1.17	−0.25	−0.12	0.44	−0.22	**0.86**
WA5	4.18(1.75)	−1.00	−0.27	−0.03	0.52	−0.24	**0.86**

SD, standard deviation; VIF, variance inflation factor.

### Measurement model

This study followed the recommendation of Anderson and Gerbing (1988) that a complete structural equation modeling analysis should be carried out in two steps. In the first stage, a measurement model should be validated to confirm that the measurements of the potential components are satisfactory before proceeding to the second stage of structural modeling.

#### Convergent validity

Referring to Table 4, the reference scholars in this study suggested that good measurement modeling criteria should include: 1. standardized factor loadings >0.7; 2. component reliabilities >0.7; and 3. mean variance extractions >0.5 (Fornell and Larcker, 1981; Hair et al., 1998). When examined sequentially, the loading values for each construct ranged from 0.73 to 0.91, all >0.7; the CR component reliabilities ranged from 0.91 to 0.94, all >0.7; and the AVE mean extractions of variance ranged from 0.67 to 0.77, all >0.5, which means that this measurement model meets the criteria suggested by the literature. Finally, to ensure that the results of this study were not biased by multicollinearity, a Variance Inflation Factor (VIF) check was conducted, and the values of VIF among all the facets were smaller than the suggested value of 3.3 (Kock and Lynn, 2012). In other words, all the constructs in this study had convergent validity and no covariance problem.

**Table 4:** Results of the measurement model.

	Items	Loading	*t*-value	*p*-value	Cronbach’s**α**	CR	AVE	VIF
1. ICT: ICT competence								
	ICT1	0.74	15.83	***	0.88	0.91	0.67	1.00 ~ 1.05
	ICT2	0.84	33.43	***				
	ICT3	0.73	17.02	***				
	ICT4	0.90	59.88	***				
	ICT5	0.87	37.31	***				
2. EE: emotional exhaustion								
	EE1	0.81	28.31	***	0.91	0.93	0.69	1.02 ~ 1.61
	EE2	0.77	23.42	***				
	EE3	0.88	54.93	***				
	EE4	0.91	77.70	***				
	EE5	0.82	27.28	***				
	EE6	0.77	18.77	***				
3. WB: well-being								
	WB1	0.89	40.41	***	0.92	0.94	0.77	1.05 ~ 1.61
	WB2	0.91	66.19	***				
	WB3	0.93	72.02	***				
	WB4	0.83	28.39	***				
	WB5	0.82	25.33	***				
4. WA: workplace anxiety								
	WA1	0.83	29.27	***	0.91	0.93	0.74	1.00 ~ 1.55
	WA2	0.91	49.87	***				
	WA3	0.84	32.54	***				
	WA4	0.86	38.10	***				
	WA5	0.86	34.90	***				

1. CR, construct reliability; AVE, average variance extracted; VIF, variance inflation factor. 2. ICT, ICT competence; EE, emotional exhaustion; WB, well-being; WA, workplace anxiety. 3.*** *p* < 0.001.

### Discriminant validity

Referring to Table 5, analysis was performed using two sets of discriminant validity determinations, AVE open-root sign method and HTMT (heterotrait-monotrait ratio). The AVE open-root sign values in bold in the table are in line with scholars’ recommendation of Pearson’s correlation coefficients for the various constructs of the lower triangles that are larger than the lower triangles (Fornell and Larcker, 1981), and the HTMT coefficients for all the constructs all met the metrics. The HTMT coefficients for each of the triangular matrices were all <0.85, which meets the conservative criteria suggested by scholars (Henseler et al., 2015); in other words, there was differential validity among the constructs in this study.

**Table 5:** Discriminant validity for the measurement model.

	1. ICT	2. EE	3. WB	4. WA
1. ICT Competence	**0.82**	*0.24*	*0.45*	*0.14*
2. Emotional Exhaustion	−0.21	**0.83**	*0.63*	*0.65*
3. Well-Being	0.41	−0.59	**0.88**	*0.32*
4. Workplace Anxiety	−0.12	0.60	−0.30	**0.86**

The figures in bold in the diagonal direction represent the square roots of AVEs; the off-diagonal elements are the correlation estimates. The upper triangular italic is the HTMT coefficient.

**Table 6:** Research hypothesis verification.

Hypothesis		Estimate	SE	*t*	*p*	Bias-corrected 95%		*R* ^2^	f^2^	Q^2^
						2.5%	97.5%			
Direct effect										
H_1_	ICT → WA	−0.12	0.07	1.66	0.10	−0.25	0.03	0.02	0.02	0.01
H_2_	ICT → EE	−0.14	0.06	2.38	0.02	−0.24	−0.02	0.38	0.03	0.26
H_3_	ICT → WB	0.30	0.06	5.29	***	0.18	0.40	0.44	0.15	0.32
H_4_	WA → EE	0.58	0.05	11.26	***	0.47	0.68		0.53	
H_5_	WA → WB	0.08	0.08	1.08	0.28	−0.07	0.23		0.01	
H_6_	EE → WB	−0.58	0.07	8.04	***	−0.72	−0.43		0.37	
Mediation										
H_7_	ICT → EE → WB	0.08	0.04	2.24	0.03	0.01	0.15			
H_8_	WA → EE → WB	−0.33	0.06	5.85	***	−0.46	−0.23			
Control										
	D1 → WA	0.04	0.08	0.51	0.61	−0.11	0.19		0.00	
	D1 → EE	−0.04	0.06	0.59	0.56	−0.16	0.08		0.00	
	D1 → WB	0.00	0.06	0.03	0.98	−0.11	0.11		0.00	
	D2 → WA	−0.02	0.07	0.33	0.74	−0.17	0.12		0.00	
	D2 → EE	−0.08	0.06	1.46	0.15	−0.20	0.03		0.01	
	D2 → WB	−0.05	0.06	0.98	0.33	−0.17	0.05		0.00	

ICT, ICT competence; WA, workplace anxiety; EE, emotional exhaustion; WB, well-being; D1, control variable; D2, control variable. Bootstrap 5,000 times; ***p < 0.001.

#### Structural model analysis

After the above steps, it was confirmed that each construct had convergent and discriminant validity, and then the overall structural model analysis was conducted. In this study, partial least squares SEM (PLS-SEM) was used, and the standardized root mean square residual (SRMR) was 0.06, which was less than 0.08, representing a good model fit (Hu and Bentler, 1999). The normalized-fit index (NFI) was 0.84, which was considered to be a standard of 0.8 for small samples (Ullman and Bentler, 2001). Finally, the GOF (Goodness of Fit) index was calculated to be 0.45 > 0.36, which means that the model in this study had a strong fit (Vinzi et al., 2010).

#### Path analysis

In the overall hypothesized validation section, school levels were transformed into dummy variables, and the following results were obtained separately after excluding the differences between the different school levels to which teachers belonged. In the direct effect section, for Hypothesis 1, teachers’ ICT competence did not reach statistical significance on Workplace Anxiety (Estimate −0.12, *t*-value = 1.66, *p* = 0.10 > 0.05). As for the direct effect on Emotional Exhaustion, Teacher ICT Competence negatively impacted Emotional Exhaustion (Estimate −0.14, *t*-value = 2.38, *p* = 0.02 < 0.05), and Teacher Workplace Anxiety positively affected Emotional Exhaustion (Estimate 0.58, *t*-value = 11.26, *p* < 0.001); therefore, Hypotheses 2 and 4 were found to be valid, with *R*^2^ = 0.38, which is consistent with scholars’ belief that an *R*^2^ between 0.33 and 0.67 is moderately explanatory (Chin, 1998). In terms of the direct effect on Well-Being, teacher ICT competence positively increased Well-Being (Estimate 0.30, *t*-value = 5.29, *p* < 0.001), but Workplace Anxiety was not statistically significant (Estimate 0.08, *t*-value = 1.08, *p* = 0.28), while Emotional Exhaustion negatively affected Well-Being (Estimate −0.58, *t*-value = 8.04, *p* < 0.001). Therefore, Hypotheses 3 and 6 were valid, Hypothesis 5 was not valid, and finally *R*^2^ = 0.44, which is within the range of 0.33 to 0.67, and so is moderately explanatory (Chin, 1998) (see Table 6; Figure 2).

#### Mediation effect analysis

For the validation of mediation effect, this study adopted the bootstrap approach (MacKinnon, 2009). In this study, bootstrapping was conducted 5,000 times to verify the mediating effect with a bias-corrected 95% confidence interval not containing 0. The mediating effect was based on the hypothesis that teachers’ ICT will be affected by the reduction of EE on WE. Hypothesis 7 held, as teachers’ ICT had an effect on WB due to a reduction in EE (Estimate 0.08, *t*-value = 2.24, *p* = 0.03 < 0.05, CI = [0.02, 15], not including 0). Assuming that Hypothesis 8 holds, the teachers’ WA will have an effect on WB via EE (Estimate −0.33, *t*-value = 5.85, *p* < 0.001, CI = [−0.46, −0.23], no zeros included).

## Discussion

In this study, partial least squares structural equation modeling (PLS-SEM) was used to investigate how teachers’ ICT competence affects the relationship between teachers’ workplace anxiety, emotional exhaustion and well-being, the results of which are discussed below.

The association between teachers’ ICT competence and workplace anxiety in this study did not reach statistical significance and therefore Hypothesis 1 was not valid. Although previous literature suggested that most teachers had not experienced online teaching before the COVID-19 pandemic, which led to workplace anxiety (Chandwani et al., 2021), in Taiwan, due to specific environmental factors, teachers had more time to prepare for online teaching because of the later outbreak. In terms of social cognitive theory, when teachers and educational institutions saw the implementation of online teaching in other countries due to the pandemic, they started to prepare for online teaching, and started to strengthen their online teaching skills in advance of the transition to online teaching (Soto et al., 2021). Such anticipatory preparation and skill enhancement may have been the main factors that made Hypothesis 1 invalid.

Hypothesis 2 was shown to be valid as teachers’ ICT competence had a negative effect on their emotional exhaustion. Although teachers often face a number of challenges in the ICT environment, including highly dispersed workload, constant interruptions, complex and difficult tasks, and long working hours, which may increase work stress generating emotional exhaustion (Pfaffinger et al., 2020), according to Social Cognitive Theory, role modeling can facilitate learning (Myers, 2018). Observational learning occurs when teachers view their peers’ high ICT competence as role models, and through observational learning, teachers’ ICT competence improves, thereby reducing their emotional exhaustion due to the work environment during COVID-19 (Pfaffinger et al., 2020).

There was a positive effect of teachers’ ICT competence on well-being in Hypothesis 3 When teachers have sufficient ICT competence, their self-efficacy is adequate, which corresponds to previous studies which have shown that teachers’ job satisfaction and well-being can be increased when they are able to possess information and technology-related competencies (Güneş and Buluc, 2017; Kasalak and Dağyar, 2020).

For Hypothesis 4, teachers’ workplace anxiety was positively affected by emotional exhaustion. In this study, teachers’ workplace anxiety positively affected their emotional exhaustion. This occurred in particular in the context of the pandemic, during which teachers faced longer working hours, high stress, lack of support from colleagues, and various factors which led to anxiety and emotional exhaustion and even mental health related problems at work (Ghasemi, 2022; Lainidi et al., 2023; Siddiqui et al., 2023).

From the results of this study, it is clear that Hypothesis 5, which suggested that teachers’ workplace anxiety would directly affect their well-being, was not statistically supported. However, the role of emotional exhaustion as a mediating variable was clear in Hypothesis 8. Even though it was previously thought that teachers’ workplace anxiety might have a direct negative effect on well-being, it is clear from this study that the effect of workplace anxiety on well-being is mainly mediated through emotional exhaustion. In other words, teachers’ workplace anxiety needs to be mediated through emotional exhaustion to have an effect on their well-being. Second, according to social cognitive theory, previous research suggests that teachers may be influenced by observing the coping strategies of their colleagues, which in turn affects their own levels of anxiety and emotional exhaustion (Soto et al., 2021). In other words, even though teachers may experience workplace anxiety when facing the challenges of the ICT environment, observing, learning, and discussing among colleagues can reduce such anxiety, which in turn indirectly enhances their well-being.

Hypothesis 6 was shown to be valid. Emotional exhaustion has been recognized in the past as a psychological symptom belonging to job burnout, which is a sense of depletion of emotional and psychological resources (Gutentag and Asterhan, 2022). This study’s findings were in line with past research, confirming that emotional exhaustion can positively and adversely affect teachers’ well-being (Fox and Walter, 2022).

This study validated hypotheses H7 and H8, and these results provide new perspectives for an in-depth exploration of the complex impacts of ICT on teachers’ well-being (WB) and emphasize the critical intermediary role of Emotional Exhaustion (EE). First, according to the Job-Demands-Resources Model (Demerouti et al., 2001), ICT as a resource can enhance teachers’ effectiveness and competence in teaching, but at the same time, the ability to use ICT also imposes new demands on teachers’ skills. However, at the same time, the ability to use ICT imposes new technical demands on teachers, which may become stressful and challenging for them, affecting their well-being. Moreover, the impact of ICT on teachers may be double-edged (Califf et al., 2020). Previous research has found that staff who perceive themselves to be less ICT competent are more likely to experience significant emotional depletion, which can affect job satisfaction and jeopardize physical and mental health (Ninaus et al., 2021).

Similarly, this study found that when teachers perceive a lack of ICT competence or lack the necessary technical support, ICT use may become a source of stress, triggering emotional exhaustion and directly impairing teachers’ well-being. Furthermore, previous research has found that teachers feel anxious at work during the pandemic for a variety of reasons (including changes in teaching styles, demands on teachers’ ICT competencies, and many others), and that workplace anxiety can lead to emotional exhaustion and further adversely affect teachers’ well-being (Vargas Rubilar and Oros, 2021). The present study found that although workplace anxiety was not found to have a significant direct effect on teachers’ well-being, workplace anxiety indirectly affects teachers’ well-being by prompting them to go through a process of emotional exhaustion. The mediating role of emotional exhaustion between workplace anxiety and teachers’ well-being emphasizes the need for schools and related management departments to proactively address and deal with teachers’ workplace anxiety in a timely manner, to alleviate the emotional exhaustion caused by workplace anxiety, and ultimately to enhance teachers’ overall well-being.

The results of this study indicate that the factors affecting teachers’ well-being are multidimensional, and this study provides an in-depth understanding of the relationships between teachers’ ICT competence, workplace anxiety, emotional exhaustion, and well-being, and can provide a strong basis for research and practice in related fields.

## Conclusion

This study attempted to investigate the relationship between teachers’ ICT competence and their well-being from Social Cognitive Theory using the PLS-SEM method, which considered the effects of including teachers’ ICT competence, workplace anxiety, and emotional exhaustion on well-being, and obtained preliminary results. Following is a breakdown of the theoretical and practical contributions of this study.

### Theoretical implications

First, at the theoretical level, this study revealed that teachers’ ICT competence was able to reduce their emotional exhaustion and enhance their well-being during the pandemic. Although ICT competence has been shown to promote well-being, it does not appear to be a major contributor to workplace anxiety during the pandemic. Furthermore, our study found that teachers’ workplace anxiety did not directly affect their well-being, but affected it indirectly through emotional exhaustion. This finding suggests that emotional exhaustion is a crucial mediator affecting teachers’ well-being. Finally, this study not only provided an understanding of the mental health and well-being of educators during the pandemic, but also found that the factors affecting teachers’ well-being were multidimensional, which will be important for the future in order to achieve the third of the Sustainable Development Goals (SDGs) of 2030 (SDG3), which aims to achieve good health and well-being for the whole person, as well as to promote education for all. This lays the foundation for more diversified choices of strategies to promote educators’ well-being in order to achieve SDG3.

### Practical implications

In terms of the practical contributions of this study, firstly, teachers’ ICT skills have some positive effects on enhancing their well-being and reducing their emotional exhaustion. This recommends that enhancement of ICT skills training in schools and education departments can help teachers better familiarize themselves with the challenges of the current educational environment. This study found that teachers’ well-being was not directly affected by their workplace anxiety, but it could have an effect by increasing their emotional exhaustion. This suggests that in order to enhance teachers’ well-being and productivity, schools and educational policy makers should consider developing appropriate intervention strategies to reduce teachers’ workplace anxiety and emotional exhaustion. Finally, this study shows that there is a positive association between ICT skills and well-being in order to achieve the third of the 2030 Sustainable Development Goals (SDG3), which is to achieve good health and well-being for all. It also gives the Ministry of Education more direction for practice; for example, the ministry can consider creating a series of teacher empowerment workshops to enhance teachers’ ICT skills and knowledge, which would not only provide appropriate support to reduce their emotional exhaustion, but also indirectly enhance their sense of well-being.

### Limitations and future study

There are several major limitations of this study: first, the completeness of the research model needs to be improved, and it has not yet covered all the variables that may affect teachers’ well-being, such as teaching experience, teaching strategies, teacher-student interactions, work environment, or school culture. Second, the representativeness of the sample was limited, focusing mainly on the southern region of Taiwan, and the convenience sampling method was used to collect data, with limitations in the geographical distribution of the sample and the types of schools. Furthermore, the study design used a cross-sectional study, which did not allow for the identification of causal relationships among variables, and the external validity and generalizability of the findings may have been affected due to the specificity of the pandemic. Finally, although this study used a scale with good reliability and validity, there is still a possibility of self-reporting bias, and there may be differences in teachers’ understanding of the scale questions in different cultures.

To address the above limitations, future research could consider the following: first, based on the existing model, explore more variables that may affect teachers’ well-being to build a more comprehensive theoretical model. Second, the study sample should be enlarged to cover teachers from different regions, urban and rural areas, school levels and types to enhance the representativeness and external validity of the findings. In addition, a longitudinal research design was adopted to collect data at different points in time to explore the dynamic changes among the variables and their mutual influence. Furthermore, a cross-cultural comparative study must be conducted to investigate the impact of cultural differences on the findings of the study and to provide a more diversified theoretical perspective. Finally, we will design and implement targeted interventions, such as ICT competency training for teachers, stress management and emotion regulation training, etc., and evaluate the effectiveness of the interventions to improve the theoretical model and provide practical guidance. At the same time, we will expand the research methodology by combining quantitative and qualitative research methods to obtain richer and more in-depth data to comprehensively understand the internal mechanisms and factors affecting teachers’ ICT competence, workplace anxiety, emotional exhaustion, and sense of well-being.

## Data availability statement

The raw data supporting the conclusions of this article will be made available by the authors, without undue reservation.

## Ethics statement

Ethical review and approval was not required for the study of human participants in accordance with the local legislation and institutional requirements.

## Author contributions

B-CC: Formal analysis, Writing – original draft. Y-TW: Formal analysis, Writing – review & editing, Writing – original draft. Y-TC: Methodology, Writing – original draft.

## Appendix

Research questionnaire.

**Table:**

Variable name	Questionnaire item
ICT competence	1. I feel comfortable using digital devices that I am less familiar with. 2. If my friends and relatives want to buy new devices or applications, I can give them advice. 3. I feel comfortable using my digital devices at home. 4. When I come across problems with digital devices, I think I can solve them. 5. If my friends and relatives have a problem with digital devices, I can help them.
Workplace anxiety	1. I am overwhelmed by thoughts of doing poorly at work. 2. I worry that my work performance will be lower than that of others at work. 3. I feel nervous and apprehensive about not being able to meet performance targets. 4. I worry about not receiving a positive job performance evaluation. 5. I often feel anxious that I will not be able to perform my job duties in the time allotted.
Well-being	1. I have felt cheerful and in good spirits. 2. I have felt calm and relaxed. 3. I have felt active and vigorous. 4. I woke up feeling fresh and rested. 5. My daily life has been filled with things that interest me.
Emotional exhaustion	1. My job makes me exhausted. 2. After I finish my job, I feel exhausted. 3. When I think of the whole day’s work in the morning, I feel tired. 4. I feel exhausted because of my job. 5. For me, working the whole day with others makes me nervous. 6. Working with others gives me lot of stress.
